# “And I Can Remind Myself That I Am All Of This”: adolescents’ experiences of group-based acceptance and commitment therapy

**DOI:** 10.3389/fpsyg.2024.1458421

**Published:** 2024-10-10

**Authors:** Filippa Brovold, Nina Jakhelln Laugen, Torun Grøtte

**Affiliations:** Department of Psychology, Norwegian University of Science and Technology, Trondheim, Norway

**Keywords:** acceptance and commitment therapy, qualitative study, group therapy, adolescents, values

## Abstract

**Introduction:**

A growing body of literature supports the use of Acceptance-and commitment therapy (ACT) for a wide range of mental health problems in children and adolescents, but less is known about ACT when given to adolescents in a group format. Consequently, this study aimed to explore the subjective experiences of adolescents who had completed a group-based ACT for symptoms of anxiety and depression. Adolescents’ perceptions of the core therapeutic processes of ACT and the means used to enhance them, as well as the interplay between ACT processes and the group format, were of primary interest.

**Methods:**

Semi-structured qualitative interviews were conducted with seven adolescents, of which five were girls and two were boys, between 16 and 19 years old. Transcripts were analyzed using reflexive thematic analysis.

**Results:**

The adolescents varied greatly in their experience of ACT’s core processes and the methods used to enhance them. Most found the core processes meaningful and educational, especially appreciating the concept of values and value-based action. However, some perceived the core processes as irrelevant and provocative, particularly interpreting acceptance as “giving up.” The metaphors and practical exercises were experiences as playful and instructive supplements to the more theoretical elements of therapy, but also as childish and embarrassing. The group format facilitated normalization and support and seemed to increase the adolescents’ motivation and adherence to therapeutical work, but it also triggered socially anxious thoughts and self-censoring for some.

**Conclusion:**

The results from the current study support previous literature indicating that group-based ACT is an acceptable and feasible treatment format for adolescents. Yet, the varying experiences underscores the need for further studies exploring how to accommodate the group format to the diverse personal and developmental disparities in this age group. Randomized controlled trials are also needed to compare the effectiveness of individual versus group format of ACT for adolescents.

## 1 Introduction

Adolescence is a vulnerable period marked by significant emotional and developmental challenges, and research indicates that the prevalence of emotional disorders (e.g., anxiety and depression) is increasing among adolescents (Mojtabai and Olfson, 2020; Vizard et al., 2020; Bakken, 2022; Thapar et al., 2022). An English survey investigating mental health among children and adolescents (5–15 years old) reported an increased rate of probable emotional disorders from 3.9% in 2004 (Green et al., 2005) to 5.8% in 2017 (Sadler et al., 2018). There are also indications of increased use of specialist mental health services in this age group (Mojtabai and Olfson, 2020). Consequently, developing and evaluating effective psychological treatments for children and adolescents, with variations in approaches and formats, is essential.

A growing literature supports the use of Acceptance-and Commitment Therapy (ACT) for common mental disorders (Fang and Ding, 2020). In 2020, Fang and Ding conducted a meta-analysis on the efficacy of ACT in children and adolescents, of where 14 randomized controlled trials (*N* = 1,189) were included. The results showed that ACT performed equally well as traditional cognitive-behavioral therapy (*SMD* = 0.3, *p* = 0.084) and outperformed waitlist condition (*SMD* = −0.86, *p* < 0.001) and other conventional psychotherapies (*SMD* = −0.59, *p* < 0.001) in terms of improving symptoms of anxiety and depression. Thus, Fang and Ding (2020) recommended ACT for use in the treatment of anxiety, stress, depression, and other mental symptoms and behavioral problems of children and youths (Fang and Ding, 2020).

**Figure 1:**
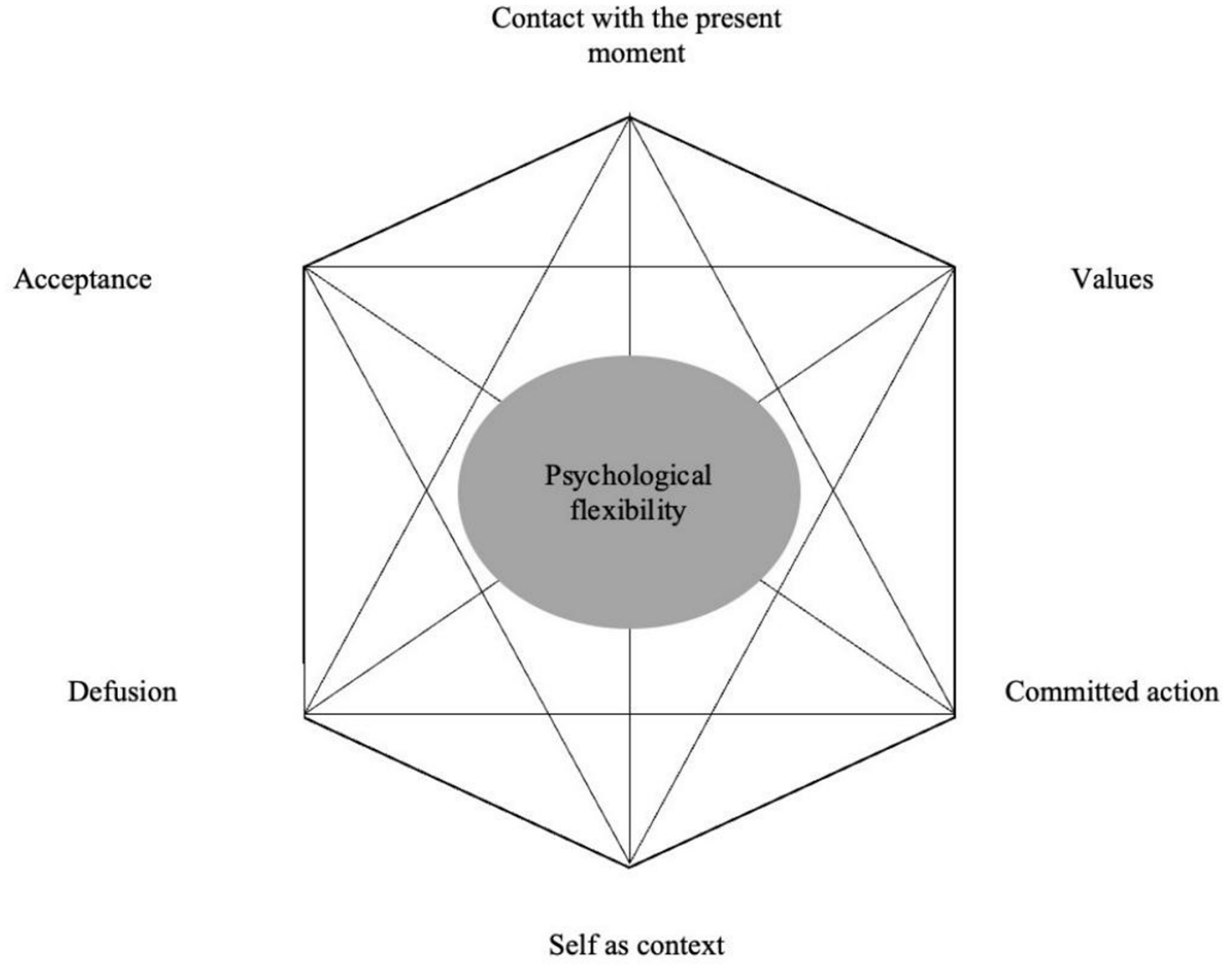
The “Hexaflex” of ACT, adapted with permission from Hayes et al. (2006).

A central term in ACT is psychological flexibility, which involves the ability to interact with internal and external events in a way that aligns with one’s values and by changing or maintaining behavior that serves valuable goals (Hayes et al., 2006; Hayes et al., 2012). Psychological flexibility is established through six core therapeutic processes, commonly represented as an interconnected hexagon, popularly called the “Hexaflex” (Hayes et al., 2006) (Figure 1). *Acceptance* entails actively embracing internal mental experiences (e.g., feeling anxious) without trying to control, avoid, or change their frequency or form (Hayes et al., 2006; Hayes et al., 2012). *Cognitive defusion* is a technique aimed at increasing the distance to one’s thoughts and feelings, where the goal is to see thoughts and feelings as “just” thoughts and feelings rather than true or accurate interpretations of reality. ACT focuses on *present-moment experiences* involving non-judgmental contact with psychological and environmental events as they occur. *Self-as-context* aims to shift individuals’ identification with an experience, emphasizing that the individual is not equivalent to their thoughts but rather a product of the context in which thoughts arise. This perspective makes it possible for the individual to create distance from painful thoughts. *Values* are chosen qualities that is supposed to motivate long-term functional behaviors. Patients are encouraged to choose life directions based on personal values while discouraging avoidance or compliance-based choices. ACT also promotes *committed action* patterns aligned with chosen values. This involves persistence and change, whichever is called to live according to one’s values in specific contexts (Hayes et al., 2006; Hayes et al., 2012).

The high dropout rate among adolescents seeking help for mental health problems (de Haan et al., 2013; Warnick et al., 2014; Gearing et al., 2012) indicates that effective treatment protocols are not sufficient in themselves. There is a need to develop interventions that adolescents find acceptable, feasible, and engaging. Adolescents’ therapists should also be aware of potential “hindering events” (e.g., difference in opinions between patient and therapist), which might negatively affect the therapeutic relationship and the adolescents’ motivation and interest in the therapy (Calabrò et al., 2024). As such, qualitative approaches are suitable to assess ACT’s acceptability and feasibility among adolescents, as it allows a more in-depth exploration of the adolescents’ experiences, needs, and preferences regarding mental health care.

So far, three qualitative studies have investigated how children and adolescents experience ACT. First, Kanstrup et al. (2019) interviewed four adolescents (12–18 years old) and four parents about their experiences of ACT for pediatric chronic pain, where the participants had received ACT as part of a group (*n* = 6) or as individual treatment (*n* = 2). Second, Ma et al. (2023) explored five adolescents’ (12–18 years old) experiences with ACT for depression, given as individual treatment. Third, Clery et al. (2021) interviewed 12 adolescents (10–17 years old) with chronic fatigue syndrome, 11 parents, and seven healthcare personnel about the acceptability and feasibility of ACT. It should be noted that none of the participants in Clery et al. (2021) had carried out the treatment themselves. As such, their perception of ACT was based on a standardized written and oral explanation of ACT, where the key elements of ACT and how it differed from traditional cognitive-behavioral therapy were highlighted.

In all three studies, adolescents described ACT as an acceptable and feasible treatment for their mental health symptoms (Kanstrup et al., 2019; Clery et al., 2021; Ma et al., 2023). Several of the six core processes of ACT were described as appealing and meaningful to work with. To exemplify, identifying, and formulating core values provided clarity and direction beyond their symptoms and challenges and was perceived as helpful in guiding, motivating, and encouraging behavior change. Acceptance was perceived as an appropriate approach for managing loss and grief as it provided compassion and stressed that thoughts and feelings were valid and grounded in actual events or understandable anxieties, not something they needed to change (Clery et al., 2021). The adolescents’ perception of cognitive diffusion seemed to vary more. Some experienced it as a helpful tool for dealing with negative thoughts, while others felt that dismissing thoughts was too difficult (Clery et al., 2021).

Likewise, there was variation in how participants experienced the form and function of ACT exercises, e.g., value clarification exercises like “the funeral exercise” or mindfulness exercises like “silent connections” (Kanstrup et al., 2019; Ma et al., 2023). Some perceived the exercises positively, highlighting that they were meaningful and gave them a more complex understanding of the principles promoted through therapy. The exercises also provided inspiration and transferability from therapy to real life and enabled the participants to adapt to new strategies when facing difficulties. On the other hand, others perceived them as unclear, too challenging, or childish. Kanstrup et al. (2019) argued that this disparity in experiences illustrates the complexity of adjusting ACT exercises to developing adolescents, who vary more widely than adults in cognitive and social functioning. They hypothesized that ACT delivered in a group format may further increase this challenge.

### 1.1 The present study

In summary, previous research on ACT for children and adolescents indicates that ACT might be an effective, acceptable, and meaningful treatment for depression, anxiety, and behavioral problems in this age group (Kanstrup et al., 2019; Fang and Ding, 2020; Clery et al., 2021; Ma et al., 2023). However, the majority of previous quantitative and qualitative studies addressed individual therapy, which means that there is still a lack of knowledge about the efficacy and experience of other formats of ACT for children and adolescents. As 12 out of 14 ACT studies in the meta-analysis by Fang and Ding (2020) were in individual treatment format, the authors argued that alternative delivery formats should be developed and evaluated also for children and adolescents.

Given the increasing incidence of mental health problems and help-seeking among adolescents (Mojtabai and Olfson, 2020; Thapar et al., 2022), group therapy may be a cost-effective alternative to individual therapy (Bjerke et al., 2021). Group therapy might also be especially beneficial among adolescents as it facilitates togetherness and belonging to peers, which are crucial developmental tasks for this age group, but some challenges are also associated with the format, e.g., self-censoring, tailoring therapy to individual needs, etc. (Gjesvik et al., 2018; McPherson et al., 2020). Moreover, the interpretation of the core therapeutic processes and the means used to enhance them can be experienced differently in a group vs. individual, e.g., carrying out practical exercises in a group compared to doing them alone.

Consequently, this qualitative study aimed to explore the subjective experiences of adolescents who had completed a group-based ACT for symptoms of anxiety and depression. The adolescents’ perceptions of the core therapeutic processes of ACT and the means used to enhance them (e.g., practical exercises, homework), as well as the interplay between ACT processes and the group format, were of primary interest.

## 2 Methods

### 2.1 Participants and procedure

The current study is based on semi-structured interviews of seven adolescents who had completed an ACT-based group treatment for symptoms of anxiety and depression. The adolescents were recruited from an outpatient clinic in the specialized child and adolescent mental health services, which is part of the public health care system in Norway. The treatment was primarily directed toward adolescents aged between 14 and 18 who were diagnosed with a depressive episode or major depressive disorder. However, comorbid mental health problems (e.g., anxiety and rumination) and moderate functional impairments such as social absence and social isolation were common. Adolescents with comorbid bipolar disorder, severe personality issues, social phobia, and substance abuse were excluded. The inclusion criteria for the current qualitative study were as follows: (1) Adolescents aged between 14 and 18; (2) Completed the ACT-based group therapy.

Adolescents were recruited from two separate ACT groups, the first was conducted during spring 2021 and the second during spring 2022. The adolescents who participated in the first ACT group were informed about the study by phone by their former therapist, whereas the adolescents who participated in the second ACT group were informed by their therapist in the penultimate group session. The first author (FB) subsequently contacted the adolescents who had verbally expressed their interest in participating and consented to be contacted. Information about voluntary participation, researchers’ confidentiality obligations, and assurance that their decision to participate or not would not impact their treatment, was provided orally and in writing. The study was approved by the Regional Committee for Medical Research Ethics (REK-ID: 308116) and the Norwegian Center for Research Data (NSD-ID: 543456). All adolescents signed a written consent form prior to participation.

Six adolescents initially participated in the first ACT group, whereof four completed it. In the second group, all eight adolescents completed. Thus, seven out of 12 possible group therapy completers opted to participate in the current study, of which three were recruited from the first group and four from the second. The final sample consisted of two boys and five girls aged between 16 and 19.

### 2.2 Treatment

The group treatment was based on the workbook “Your Life, Your Way: Acceptance and Commitment Therapy Skills to Help Teens Manage Emotions and Build Resilience” (Ciarrochi and Hayes, 2020). The overarching goal was to help adolescents create new and adaptive ways to deal with difficult emotions and thoughts. Five core therapeutic processes from ACT were introduced and practiced: acceptance, cognitive defusion, contact with the present moment, values, and committed action. Self-as-context was excluded by decision of the therapists, due to the level of difficulty and abstract understanding needed for this core process, which might not be achieved for adolescents between the ages of 13–18 in such a short time frame.

To enhance the core therapeutic processes and promote the integration of ACT principles into daily life, metaphors, practical exercises, and homework were used (Hayes et al., 2006; Hayes et al., 2012). Some examples include the “Tug-of-War with a Monster,” where patients are asked to imagine their fears and doubts as a monster with which they are in a tug-of-war and then reflect on what will happen if they put down the rope (Ciarrochi and Hayes, 2020). This exercise is designed to promote acceptance and help the group member realize that there are better options than fighting their thoughts and feelings. Another example includes the “Funeral Exercise,” which is about imagining your funeral and what you think and would like people to say about you. The goal is to reflect on and clarify values. Homework was given between each group session. Examples include reflection on values and creating behavioral goals aligning with them, promoting committed action. Other essential treatment components included psychoeducation and validation. Additionally, the adolescents’ relatives received information about the treatment to encourage and enable the relatives to help their adolescents incorporate ACT principles into their everyday lives.

The first group was led by one therapist and the second by two. All therapists were clinical psychologists. The initial ACT group comprised six treatment sessions lasting 2.5 h each. Drawing from insights from the first group, modifications were implemented for the second group. This involved changing the number of sessions to three, each lasting 2.5 h, with individual treatment sessions provided between each group meeting. In sum, the group treatments consisted of fewer, but longer sessions than typical individual treatments, as previous research and clinical practice indicate that ACT usually involves 8–12 sessions ranging from 45 to 150 min (Ong et al., 2018; Bai et al., 2020).

### 2.3 Data collection

Semi-structured qualitative interviews exploring the adolescents’ experience participating in group-based ACT were undertaken between May and June 2022, each lasting 45–60 min. The main topics in the interview guide (see Supplementary material) were adolescents’ experiences with the group format, the core therapeutic processes, and experiences of change in mental health symptoms and daily functioning. Questions were open-ended and exploratory. Follow-up questions were used to elaborate on specific topics or check the interviewer’s understanding of the adolescents’ experiences, allowing them to validate, clarify, or elaborate on their statements.

A fifth-year clinical psychology student (FB) conducted the interviews. In accordance with recommendations from the ethical committee, a clinical psychologist (TG) was also present to ensure interview quality and care for the adolescents. The interviewers had no previous relation to the participants. All interviews were conducted digitally through Confrere, a secure video call service with end-to-end encryption, meeting the Norwegian authorities’ security requirements (e.g., Normen and General Data Protection Regulation). All interviews were audio recorded and transcribed verbatim by FB.

### 2.4 Data analysis

Data were analyzed using reflexive thematic analysis (Braun and Clarke, 2022). Reflexive thematic analysis is a flexible, yet systematic approach to data analysis, valuing the researchers’ subjectivity as the primary way to discern meaning from the data. This approach was chosen, as the flexible application of thematic analysis allowed the researchers to apply ACT theory as the theoretical framework and for the analysis to be informed by ACT’s six core processes. The researchers involved in the analysis were two experienced clinical psychologists (TG and NJL) and a fifth-year clinical psychology student (F.B.). Although none of the researchers had formal training in ACT, FB had a strong interest in this treatment method, and TG, and NFL were formally trained in other forms of cognitive and third-wave cognitive therapies. A phenomenological-hermeneutic approach was employed in the analysis (Ho et al., 2017). The aim was to capture the adolescents’ experiences reflected in the interviews, while also recognizing and allowing the researchers’ previous knowledge and professional background to shape the interpretation of the data.

Although the analytic process alternated between individual text analysis and group discussions, the general sequence of the analysis was as follows:

1. To familiarize themselves with the data, the researchers (FB, TG, and NJL) read the transcripts individually several times.
2. Preliminary codes were drafted. A deductive (top-down) approach to coding was used, as ACT principles served as a theoretical framework from which data were analyzed. However, the coding also had inductive, semantic, and latent elements.
3. The team discussed the codes to create a richer and more nuanced understanding of the data. Group discussions contributed to increased reflexivity, as they allowed the researchers to examine their assumptions and how these influenced the interpretation of the data. It was agreed to narrow the research question to the adolescents’ experiences and interpretation of the therapeutic core processes and means of ACT (form and function of the therapy intervention), as well as the interplay between ACT processes and the group format. Based on this, transcripts were reread, and codes were revisited and adjusted accordingly.
4. Looking at the meaning units across interviews, candidate themes were generated, reflecting what emerged as the most critical aspects of the adolescents’ converging and diverging experiences.
5. Through discussion between the researchers, themes were further elaborated and adjusted to ensure that the meanings relevant to the research question were captured.
6. The original transcripts were revisited to check that all relevant aspects of the adolescents’ experiences had been included in the analytic process. Subsequently, the final themes and sub-themes were determined.
7. A description of each theme was written up, and sample quotes capturing the core of each theme were chosen. To ensure the anonymity of adolescents, pseudonyms have been used throughout the results section.

## 3 Results

**Figure 2:**
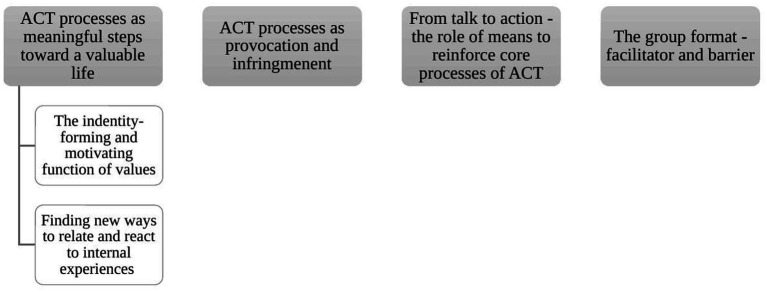
Themes and subthemes.

The analysis yielded four superordinate themes: (1) “*ACT processes as meaningful steps toward a valuable life*”; (2) “*ACT processes as provocation and infringement*”; (3) “*From talk to action – the role of means to reinforce core processes of ACT*”; and (4) “*The group format – facilitator and barrier*.” The themes (see Figure 2 for a presentation of themes and subthemes) capture the adolescents’ highly varying experiences and perceptions of the core therapeutic processes of ACT and the practical exercises (e.g., homework), and the interplay between ACT processes, the means, and the group format.

### 3.1 ACT processes as meaningful steps toward a valuable life

This theme concerns the ways the adolescents found the core therapeutic processes of ACT meaningful and applicable to their personal lives and emotional challenges.

#### 3.1.1 The identity-forming and motivating function of values

Five adolescents stated that the therapy helped them to understand their values better. The ACT-group provided a safe environment for reflecting upon which values were important to them and how these values could help them in their everyday lives. Thus, the therapeutic work with values seemed to serve several functions for them.

First, the adolescents considered values as appreciated personal qualities. Through the therapy, they became more aware of which values were essential in their lives (e.g., authenticity, independence, and honesty), and by this, they were also more conscious of what they valued about themselves. They highlighted that these qualities were something they could be proud of and that they played a crucial role in making them who they were. As such, it seemed like values had an identity-forming function.

Moreover, as highlighted in the following quote by Sophia, this identity-forming function of values seemed particularly important during “dark times.” Due to their mental health problems, the adolescents’ minds had been preoccupied with negative thoughts about themselves and worries about the future. Thus, the ACT group’s focus on values seemed to help the adolescents understand that they were more than their mental illness, and that their psychological problems and life difficulties could coexist with their valued personal qualities.

*And I can remind myself that I am all of this and can be so proud of myself because I have all these good values… When you are in a bit of a dark place, I feel that all the bad things become, in a way, the only thing you see and the only thing you hear in your head…But to talk with the other ones in the group, saying but I have all these good values… It was good to say it out loud and speak well of yourself when things are kind of dark and sad (Sophia)*.

Second, the adolescents highlighted that values could guide their lives. When faced with something difficult, they could remind themselves of their values (i.e., who they were and what they wanted in life) and use this to figure out how to get better. In this way, values seemed to function as a motivation and inspiration for adaptive and health-promoting actions. In the following quote, Jacob describes how the therapeutic work with his values “honesty” and “openness” helped him to implement actions that were more in line with his values.

*When I was in a terrible place [before therapy], one of my biggest problems was that I isolated myself…I was not honest and open with the people I needed to be honest and open with… If only I had been honest and open about everything happening to me and bothering me, it would have been so much easier to solve it and live better… [after therapy], I have tried to talk very openly about my thoughts and feelings with people close to me.*

Importantly, the adolescents also emphasized that it is not sufficient to just to talk or think about one’s values, but that one must carry out actions and behavior that align with one’s values and serve valued goals. Several adolescents reflected to a greater extent on their actions in light of their values after the ACT group. Although adolescents found it challenging to act according to their values (e.g., easier to continue to do things “the old way”), the treatment inspired them to attempt new value-based changes and actions in their lives. Casper highlights this:

*Values do not help us if they are only in our minds because they should also turn into action, such as creativity. If I sit and think I want to be creative, I do not get anything out of my value*.

#### 3.1.2 Finding new ways to relate and react to internal experiences

Prior to treatment, most adolescents described being very caught up in what was happening inside their heads, i.e., they spent much time focusing on their negative thoughts and feelings. As an attempt to control or remove these negative thoughts and feelings, all adolescents frequently used maladaptive coping strategies such as worry, rumination, avoidance, and distraction (e.g., keeping themselves occupied with social media or constantly being with others). This way of living made them feel disconnected from the real world around them:

*For such a large part of all the negative thoughts is that everything is somehow in one’s head, and then one lives in one’s head. And then, in a way, you are not living in the real world. You are living in your head (Jacob)*.

Five adolescents described how their understanding of, and their ways to relate to, their emotional symptoms and problems in everyday life changed during treatment. They realized that it was possible to relate to internal and external events in an alternative way, i.e., by accepting them without any attempts to control, avoid, or change them. Although there was some discussion in the group about what acceptance of your problems means (see theme 2 for elaboration), most adolescents stated that they learned that painful thoughts and feelings are ordinary, human, and transitory, and therefore something they should register and accept rather than fight against. They were left with the idea that it is possible to acknowledge negative thoughts and feelings without letting them overwhelm one’s mind, and that this way of responding would allow them to focus on and use their energy on other important things in their lives:

*We had to accept it to sort of say, okay, this is happening. […] And it made me, in a way, register that you do not have to stand and pull on that rope; you can leave it there and go do something else (Casper)*.

Adding to their new discovery about acceptance, adolescents also described that they became aware of the possibility of diverting one’s attention away from worries and rumination in a more adaptive way. Rather than trying to change thoughts or feelings in themselves, they realized that they could change how they reacted to them. They discovered that they had a choice—that they had the ability to take control over the way they related to negative thoughts and feelings by actively deciding not to pay undue attention, listen to, or engage with them. Putting the negative thoughts aside or creating distance from them is possible.

*So, then it was almost like that, in a way, trying to put the negative thoughts to the side. Because they do not just go away, but [you can choose] to not quite listen to them (Sophia)*.

Some adolescents also described that the treatment made them realize that they should not allow negative thoughts and feelings to limit their actions. E.g., anxiety and fear should not lead to avoidance of the feared situations, but instead be handled with confrontation of the things they were afraid of:

*I remember we did an exercise about continuing to do things you are afraid of, even if you have many thoughts that try to pull you back… And in a way, accepting it… It was about accepting that the thoughts are there. However, you can still do something about what you fear or struggle with (Hanna)*.

By doing so, the control that the negative thoughts and feelings had over the adolescents was reduced, thereby allowing them to be more present in the real world.

### 3.2 Theme 2: ACT process as provocation and infringement

Although several adolescents experienced the core processes of ACT as helpful and meaningful (*cf.* Theme 1), there was substantial variability in how these core processes were perceived. Two adolescents stated that the therapy did not help them as much as they had hoped beforehand and attributed their lack of change to aspects of the treatment. Thus, theme 2 is concerned with the ways that adolescents perceived the core processes of ACT in a more negative way, e.g., as provocative and offensive.

The core therapeutic process “accept” seemed to be one of the most challenging aspects of the treatment. Some adolescents interpreted the therapeutic work with acceptance as an encouragement to preserve, persist, and stand in the face of adversity. The way that acceptance was presented and portrayed as a “solution” to the adolescents’ difficulties evoked strong emotional reactions among some. They referred to it as “*provocative*,*” “offensive*,*” “strange*,*”* and *“stupid*.*”* Emma described this as follows:

*It was really to sort of accept that things are as they are. I felt that everyone was provoked in the group… It was actually very annoying to sit and listen to… [Imitating the therapist with an exaggerated voice] “You have to accept that you have a monster in your backpack” … It was actually quite provocative … You have to accept that [your] life is shit. I found that a bit silly.*

Some of the adolescents seemed to interpret “accept” as the equivalent of giving up hope for a better life. This was perceived as terrifying because they imagined a life with as much pain and struggle as the life they were living now. They felt that by listening to the suggestions from the treatment, they would remain stuck where they already were without the prospect of a better life. Some also highlighted that they previously had tried to accept that things were painful and difficult but that there is a limit to how long one can continue doing this, and implicitly that, for their part, they could no longer accept the status quo. In other words, there was an “acceptance” limit. Leah:

*We talked a lot about accepting that we are struggling…I remember reacting very strongly to that. Because I feel it is a bit wrong to accept that you are struggling and then continue with your life… One of the therapists said that you should accept that you are struggling, stick to your values, and look ahead in life. It will become a heavy backpack but hold on to it and keep going up the mountain. And then I felt that, yes, you can manage to go up that mountain with a heavy backpack for probably a few months or years, but you reach a breaking point. I have experienced that. I have accepted that I felt the way I did, but I reached a breaking point*.

The core process of “values” also seemed to be challenging for some of the adolescents. Some described that it was difficult to see the relevance of values to their own challenges, as well as understand how values could help them resolve their issues and move forward. Although several highlighted that they did grasp the theoretical concept of values, they struggled to establish a tangible connection between values and their current circumstances. For these adolescents, the suggestion to “turn to their values” seemed inadequate in addressing the complexity and difficulty of their present problems. This disconnection caused frustration and confusion.

*They mostly talked about us being “stuck” and that we had to hold on to our values. They talked a lot about values and things like that in general. I understood the concept, but it does not help me exactly where I am in my life right now (Leah)*.

### 3.3 Theme 3: from talk to action—the role of means to reinforce core processes of ACT

As previously mentioned, metaphors, practical exercises, and homework were frequently used in group sessions to enhance the core therapeutic processes and promote the integration of ACT principles into daily life (Hayes et al., 2012; Hayes et al., 2006). The third theme concerns the variation in how the adolescents experienced and interpreted the form and function of the more “practical” and active parts of ACT. All seven adolescents contributed to this theme.

The variation in attitudes was particularly evident toward homework, which was given to all adolescents between each group session. Some expressed that they experienced the homework assignments as challenging but advantageous, as the challenging nature of the tasks facilitated reflection on the topics covered in therapy:

*And sitting down and thinking about it and writing about it, I think that was very nice. When I first heard about homework, I thought, “Oh, even more schoolwork than I already have.” But I found it nice to sit down and reflect (Sophia)*.

Others pointed out that homework helped them to transfer what they learned in therapy to their own lives.

*We could reflect on what we had worked on between sessions (…) Then, in a way, you were able to use what we learned and put it into your own life (Hanna)*.

However, there were also some adolescents that perceived homework in a more negative way. Some reported that they did not receive a satisfactory explanation that would enable the implementation of homework, which reduced their motivation to do the task. Others pointed out that they felt that homework was merely a repetition of the topics discussed during group sessions. They could not see how the homework added anything new and valuable to them and therefore saw no point in doing it. Leah sums this up:

*After each time, we got a paper that we had to fill in and do stuff like that at home … I did not really bother … Because we also did it in the group. So, I felt it became a bit like I had to go home and complete it at home, too … When I had, in a way, [already] done it in the group*.

The practical exercises were also perceived differently among the adolescents. Some highlighted that they helped create an informal and relaxed atmosphere in the group sessions because they were fun and playful. They made the therapy feel less “*therapy-like*.” It was also stated that it was easy to understand the purpose of the tasks and their relevance to the treatment content, and that the tasks helped them understand the core processes of ACT even better. To exemplify, Sophia describes how a specific practical exercise helped her reflect on how values could guide her actions and how she could handle her negative thoughts in the future:

*So, there was this group behind her, saying all these hurtful things while she was trying to move toward the good things [values] … It was kind of painful and heartbreaking to scream at someone and say, “You are not good enough.” … But in a way, we got a glimpse of her moving toward something and trying to set aside those negative thoughts … And I feel like I can also set aside my own [negative] thoughts*.

Another adolescent (Jacob) referred to the same practical exercise as Sophia. He reflected on how the experience of saying negative things out loud to the group members highlighted the absurdity of the negative things they often said to themselves. By doing this exercise, he was able to create some distance to his negative thoughts, realizing that they might be false, unimportant, and not helpful. Thus, the practical exercise seemed to increase his understanding of the core process of cognitive defusion:

*It was kind of strange … It was a bit odd actually to say it to someone's face… Because you, or at least everyone in the room, were quite used to hearing it from themselves. Inside their heads… It provided a bit more reassurance that listening to such things from oneself is not something one should do… When I said it to someone else, I had the feeling that this is nothing and does not help at all (Jacob)*.

Moreover, several adolescents highlighted that the insight they gained from these practical tasks was different than a more theoretical understanding of the core processes of ACT. Understanding therapy principles intellectually is one thing, but experiencing the process is different. Thus, most adolescents perceived the practical tasks as instructive and necessary supplements to the more “theoretical” psychoeducation and group conversations.

However, the positive view of the exercises was not shared by everyone, as some experienced the tasks as childish and unpleasant. Others perceived the exercises as unnecessary therapeutic elements, as they felt that the tasks did not help them or give them a different understanding of the core processes of ACT than the ordinary group conversations did:

*I thought the exercises could be a little childish. However, that is how I felt … I did not really need to do these exercises … I think talking about it was just as good (Sophia)*.

*The exercises were a bit weird … it was a bit embarrassing. Because, after all, we are not children” (Emma)*.

### 3.4 Theme 4: the group format—facilitator and barrier

Theme 4 deals with the adolescents’ experiences of being part of a group, and how the group format worked as a facilitator and barrier for dissemination of the therapeutic content and engagement in treatment. All seven adolescents shared their opinions on this, and they seemed to appreciate the group format for several reasons. During therapy, a sense of community and belonging to the group was developed. The opportunity to meet adolescents who also were struggling with emotional problems contributed to a normalization of difficulties, a weakening of their previous feeling of “being alone.”

*It was very nice to hear that others can relate to what I [feel], even if it may not be exactly the same situation, but somehow a similar situation in which they have felt the same … It was like, okay, others experience this too. I am not completely alone. Even though I kind of knew that before, [now] I can put a face on a person who also feels that way (Casper)*.

The adolescents also appreciated the opportunity to reflect and exchange experiences with each other. Sharing thoughts, feelings, and experiences related to the treatment with the other group members seemed to broaden the adolescents’ perspectives and increase their motivation and adherence to therapeutic work, e.g., inspiring them to try homework or practical exercises that other group members had positive experiences with.

*And to use a book, I found it a bit silly. However, many in the group tried it, and then it became more like, okay, maybe I should also try it. So, then I tried to write down my thoughts. I tried doing the breathing exercise. Because it can be a bit silly if you only hear it from a psychologist (Anna)*.

Moreover, several adolescents described that they found it easy to relate to each other’s situation and perspectives, and that it was easier to be inspired by group members who were close to their age compared to their (older) therapist. Some also stated that they learned more by doing the practical exercises as a group rather than alone with a therapist:

*All the therapists I have talked to have been like 30 years older than me. It felt like they were in another universe. It was very nice to see people close to my age who had reached a point in their life that I wanted to achieve as well, and to physically see that it was possible … That was kind of inspiring to me (Jacob)*.

*You gained a greater understanding when you tried to explore how they [the exercises] work in a group. I noticed that it is more difficult to do an exercise [alone] with a clinical psychologist than together in a group. Like, how do you perform it? Should we do that now? You learn better and faster when you can try and fail together (Anna)*.

Although several advantages of the group format were described, the group format also brought some challenges. Some adolescents felt that it was embarrassing and uncomfortable to carry out the practical exercises in the group.

*I found it a bit awkward … I am not that comfortable with new people (Hanna)*.

Others described socially anxious thoughts, e.g., feared that their reflections or experiences would not be relevant to the rest of the group. Additionally, some adolescents had prior knowledge of other group members (e.g., attending the same school), and feared that private information would be spread. Consequently, some adolescents described that these factors placed limitations on how much they shared with the group, and that they appreciated the homework between group sessions because it became a more “private” alternative to the sharing and openness that was encouraged in group sessions.

*I felt that I couldn't say everything, even though they said it was allowed to be open. There were things I didn't want to say, which might be unnecessary and not relevant … I was like, it's not relevant, so I don't need to share that about myself. So, you open up about 1/3, I feel (Casper)*.

*And it is not something everyone finds easy to talk about, at least not out loud in front of the group. And the fact that I could write it down and reflect [by myself] was quite nice. Because, in a way, it is just me and my own thing (Sophia)*.

## 4 Discussion

The current study aimed to explore adolescents’ perceptions of the core therapeutic processes of ACT and the means used to enhance them (e.g., practical exercises, homework), as well as their experiences of being part of a group and the interplay between ACT processes and the group format. There was great variation among the adolescents regarding their perception and experience of group-based ACT. The majority experienced ACT’s core processes as meaningful and educational, particularly appreciating the concept of values and value-based action. However, some perceived the core processes as irrelevant and provocative, particularly interpreting acceptance as “giving up.” The metaphors and practical exercises were experiences as fun and playful elements that helped them understand the core processes of ACT even better, but also as childish and embarrassing. The group format seemed to facilitate normalization and support, increasing motivation and adherence to therapeutic work, while at the same time triggering socially anxious thoughts, leading to self-censoring for some.

Most adolescents in the current study found the core therapeutic processes of ACT meaningful and relevant to their lives and mental challenges. Through therapy, they understood that it is not always possible to control what is happening but rather that it is possible to acknowledge and accept it. This led to new ways of relating and responding to negative internal experiences, laying the foundation for them to make more value-based changes and actions in life. This description of symptom reduction following ACT is in line with the results of previous quantitative studies of ACT for children and adolescents, which indicated that ACT was an effective, acceptable, and feasible treatment for these age groups (Fang and Ding, 2020). It also aligns with findings from previous qualitative studies of children, adolescents (Kanstrup et al., 2019; Clery et al., 2021; Ma et al., 2023) and adults (Bacon et al., 2014; Smith, 2017; Davies et al., 2019; Fogelkvist et al., 2021; Bahattab and AlHadi, 2021; Bloy et al., 2021) of where all indicated that the six core processes of ACT were appealing and meaningful to work with and contributed to participants’ improvement in mental health and overall well-being.

In line with the study by Kanstrup et al. (2019), value-based work seemed particularly appealing to the adolescents participating in the current study. This differs somewhat from studies exploring the adult’s experiences of the core therapeutic processes of ACT. Although most qualitative studies of adults mentioned values as a helpful component in treatment, contact with the present moment was highlighted and experienced as the most critical and significant core process (Bacon et al., 2014; Davies et al., 2019; Bahattab and AlHadi, 2021). This difference may stem from methodological differences between studies, e.g., wording and choice of questions in the interview, differences in analytical approach, treatment, therapists, etc. However, this may also be explained by the fact that, unlike adults who have already gone through the critical phase of identity formation, value-based work may be especially suitable and engaging for developing adolescents. Erik Erikson’s eight-stage theory of psychosocial development describes identity versus role confusion as the fifth stage of development, which occurs during adolescence (Erikson, 1968). During this stage, people explore their independence and develop a sense of self. Moreover, research exploring how adolescents understand their values indicated a link between values and self-concept and that values could guide and motivate behavior aligned with short-and long-term goals (Lewis-Smith et al., 2021). This was highlighted by our adolescents as values seemed to have an identity-forming function in their lives. This may, in contrast, be less important to adults who often have a clearer sense of their own identity.

Although most adolescents expressed satisfaction with ACT as a treatment intervention, some held a different perspective. Specifically, these two adolescents strongly reacted to the core process of “acceptance” and, to a somewhat lesser extent, the core process of “values.” This is in line with the results of Clery et al. (2021), who revealed that some mental health care professionals had concerns about the word “acceptance” and how this core process could be easily misunderstood. Their main concern was that parents and patients might confuse “acceptance” with “giving up,” thereby finding it hard to accept a therapy advocating for acceptance. This is consistent with the current study’s findings, where some adolescents interpreted the call to “accept” as a call to give up hope for a better life, which was perceived as provocative and led to irritation and confusion. To reduce the chance of misunderstandings, Harris (2018) proposed to use the term “willingness” as an alternative to “acceptance.” While “acceptance” can be interpreted as resignation, tolerance, and “gritting your teeth and enduring,” the term “willingness” implies allowing one’s thoughts and feelings to be as they are in the present moment.

The adolescents’ challenges with “acceptance” is also consistent with previous qualitative studies of adults’ experiences with ACT, where several participants initially experienced the concept of acceptance as confusing, scary, and disappointing (Smith, 2017; Bahattab and AlHadi, 2021). However, the adults’ perception of acceptance changed throughout treatment, where most adults gained a greater understanding and acceptance of the core process of accept at the end of treatment. This emphasizes the importance of time, as some individuals may need more time to process therapeutic concepts than others. It also stresses the importance of therapists being aware of potential “hindering events,” as moments of mismatch between therapists and (adolescent) patients frequently occur (Calabrò et al., 2024). To identify and resolve these “hindering events,” Calabrò et al. (2024) recommend therapists to encourage their patients to share their doubts, fears, and uncertainties throughout therapy.

For some adolescents, the experiential exercises, homework, and metaphors used in therapy seemed to enhance their understanding, engagement, and application of the principles and skills necessary to improve psychological flexibility, consistent with the experiential learning that ACT aims to promote (Bennett and Oliver, 2019). This is in line with previous qualitative findings from studies of adults (Bahattab and AlHadi, 2021; Fogelkvist et al., 2021). However, a few adolescents in the current study found the exercises and homework confusing, overly complex, and childish, which was also described by the adolescents in the study of Kanstrup et al. (2019). Interestingly, Bacon et al. (2014) also noted that some adult participants with a psychosis diagnosis also found some of the exercises more comical than helpful. However, this was not reported by any of the other studies of adults.

As indicated by previous literature on group psychotherapy (Gjesvik et al., 2018; McPherson et al., 2020), factors common to the group format (i.e., not related to ACT specifically) were highlighted as beneficial by the adolescents. Prior to therapy, several adolescents described it as taboo, lonely, and shameful to have a mental illness. Participation in the group, however, contributed to a normalization of having emotional problems, leading to less stigma and an increased feeling of “togetherness” and social support.

The group format also seemed to influence the adolescents’ attitudes and experiences of the core therapeutic processes of ACT and the means used to address them. For some, the group felt like a safe space where it was possible to explore, reflect, and learn together, thereby leading to a greater understanding of the core processes of ACT. Joint engagement in practical exercises and sharing one’s work also seemed to increase motivation, engagement, and adherence to the therapeutic work, which are key factors in preventing drop-out, especially for children and adolescents (Gearing et al., 2012; Warnick et al., 2014). In contrast, others found the group exercises embarrassing and preferred more private ways of working with the core therapeutic processes. Although it was not said directly by the adolescents, we have the impression that some feared making a fool of themselves by participating in exercises they considered somewhat childish. Some also reported self-censoring, as they felt that their experiences or reflections were not important enough for the group. Similar findings were also reported by McPherson et al. (2020, p. 11), where patients undergoing group psychotherapy for depression sometimes found it difficult to share their problems with the group, as they saw their problems as “minor” or “not permissible.”

In summary, the various experiences of the adolescents participating in the current study underline the challenge of adapting the core processes and exercises of ACT to a developing youth population, which broadly varies in cognitive and social functioning, as problematized by Kanstrup et al. (2019). These findings indicate that this might be particularly challenging in a group format, as the therapist has less time to tailor the content to each individual’s needs. As highlighted by Fang and Ding (2020), there are currently few quantitative studies of group-based ACT for children and adolescents, which underscores the need for further studies evaluating the effectiveness of group-based ACT for this age group. In the future, randomized controlled trials directly comparing ACT in an individual vs. group format should be conducted. This would allow for a direct comparison of their effectiveness, acceptability, and feasibility, as well as each format’s ability to convey the core message of ACT. Lastly, further qualitative studies on ACT are needed to further illuminate the processes that facilitate therapeutic change in general, and particularly among children and adolescents.

### 4.1 Limitations

A few limitations should be considered when interpreting and discussing the results. First, the adolescents in the current study were recruited from two different ACT groups led by two different therapists. Although the therapists followed the same treatment manual, the two groups differed in terms of number and duration of sessions, whether or not individual treatment was added to the group sessions, and to what extent relatives were involved. It is therefore important to emphasize that the adolescents do not necessarily reflect on the same experiences. Moreover, as the current study lacked a measure of treatment fidelity, further research should explore how therapists’ adherence to ACT protocols might affect adolescents’ experiences of the core therapeutic processes of ACT.

Second, the adolescents recruited from the first group had completed therapy approximately 1 year before the interviews, making them more vulnerable to recall bias (Clarke et al., 2008). They may not remember their therapeutic experiences as vividly as the adolescents from the second group who had just recently completed therapy, which could impede their ability to engage in conversations about them.

Finally, as all adolescents were treatment completers and 16 years and older, the findings may have somewhat limited transferability to other groups, e.g., children under the age of 16 and treatment dropouts.

## 5 Conclusion

The results from the current study support the notion of group-based ACT as an acceptable and feasible treatment format for symptoms of anxiety and depression, as most adolescents found the core processes and practical exercises of ACT as meaningful and educational. The group format facilitated normalization and support and seemed to increase the adolescents’ motivation and adherence for therapeutical work. However, the substantial variation in how adolescents perceived the core processes and therapeutical means of ACT (e.g., acceptance interpreted as “giving up”) highlights one of the major challenges with the group format, as it to a lesser extent than individual therapy allows for personalized adjustments based on the individual needs of each participant. Thus, further research is needed to explore how group-based ACT could be adapted to meet adolescents’ personal and developmental differences. This includes interventions such as individual therapy between group sessions, as suggested by Gjesvik et al. (2018), and the involvement of family members, which was highlighted as important by Warnick et al. (2014). Additionally, personalized homework assignments could be implemented to address the unique needs of each group member. Further research should also examine how younger children experience ACT processes and therapeutic tools, as well as the experience of children and adolescents who do not complete ACT.

## Data Availability

The datasets presented in this article are not readily available because of consent restrictions from participants. The transcripts are only available to the authors of this manuscript, but the list of codes that was generated in the qualitative analysis is available from the corresponding author. Requests to access the datasets should be directed to torun.grotte@ntnu.no.
