# Supplementary material for: “And I Can Remind Myself That I Am All Of This”: adolescents’ experiences of group-based acceptance and commitment therapy
Source: Front Psychol. 2024 Oct 10;15:1458421. doi: 10.3389/fpsyg.2024.1458421 (PMC11500323; doi:10.3389/fpsyg.2024.1458421)
Supplement: Supplementary file 1 [file Table_1.DOCX]

Supplementary Material

**“And I Can Remind Myself That I Am All Of This”: Adolescents’ Experiences of Group-based Acceptance and Commitment Therapy**

Filippa Brovold^1^, Nina Jakhelln Laugen^1^, Torun Grøtte^1^*

^1^Department of Psychology, Norwegian University of Science and Technology, Trondheim, Norway.

*** Correspondence:** Torun Grøtte: torun.grotte@ntnu.no

**Interview Guide**

*Questions about the informant's experiences with participating in Group-Based ACT to manage depression and anxiety*

1. Tell me a bit about what it was like for you to participate in the ACT group.
2. What did you think about the content of the treatment?
3. Which themes made the most impression on you?
   1. Why?
4. How was it to complete the exercises?
5. How was it to do the homework?
6. What changes did you experience as a result of the treatment?
7. What do you think worked well with the treatment for you?
8. What did not work well with the treatment for you?
9. How did the presence of other group members affect you?
   1. Positive aspects of being in a group
   2. Negative aspects of being in a group
   3. How was it to share with the group?
   4. How was it to listen to the others in the group?
10. What did you gain from this experience?
    1. Tell me about a special event or experience.
11. Would you recommend group-based ACT treatment to others?
    1. Why/why not?
12. What can be done to make the treatment program even better?
